# Does schooling attained by adult children affect parents' psychosocial well-being in later life? Using Mexico’s 1993 compulsory schooling law as a quasi-experiment

**DOI:** 10.1016/j.ssmph.2024.101616

**Published:** 2024-02-10

**Authors:** Sirena Gutierrez, Emilie Courtin, M. Maria Glymour, Jacqueline M. Torres

**Affiliations:** a Department of Epidemiology and Biostatistics, University of California, San Francisco, San Francisco, CA, USA; b Department of Health Policy, London School of Economics and Political Science, London, United Kingdom; c Department of Epidemiology, Boston University School of Public Health, Boston, MA, USA

**Keywords:** Education, Intergenerational influences, Lifecourse epidemiology, Depression, Quasi-experimental methods, Socio-economic status

## Abstract

Higher adult child educational attainment may benefit older parents' psychosocial well-being in later life. This may be particularly important in low- and middle-income countries, where recent generations have experienced comparatively large increases in educational attainment. We used data from the 2012 Mexican Health and Aging Study, a nationally representative study of adults aged ≥50 years and leveraged the exogenous variation in adult child education induced by Mexico’s compulsory schooling law passed in 1993. We employed two-stage least squares (2SLS) regression to estimate the effects of increased schooling among adult children on parents' (respondents') depressive symptoms and life satisfaction scores, controlling for demographic and socioeconomic characteristics. We considered heterogeneity by parent and child gender and other sociodemographic characteristics. Our study included 7186 participants with an average age of 60.1 years; 54.9% were female. In the 2SLS analyses, increased schooling among oldest adult children was associated with fewer depressive symptoms (β = −0.25; 95% CI: −0.51, 0.00) but no difference in life satisfaction (β = 0.01; 95% CI: −0.22, 0.25). Stratified models indicated differences in the magnitude of association with depressive symptoms for mothers (β = −0.27, 95% CI: −0.56, 0.01) and fathers (β = −0.18, 95% CI: −0.63, 0.26) and when considering increased schooling of oldest sons (β = −0.37; 95% CI: −0.73, −0.02) and daughters (β = −0.05, 95% CI: −0.23, 0.13). No parent and child gender differences were found for life satisfaction. Power was limited to detect heterogeneity across other sociodemographic characteristics in the second stage although first-stage estimates were larger for urban (vs. rural) dwelling and more (vs. less) highly educated respondents. Results were similar when considering the highest educated child as well as increased schooling across all children. Our findings suggest that longer schooling among current generations of adult children, particularly sons, may benefit their older parents' psychosocial well-being.

## Highlights

• Growing research suggests adult child education influences older parents' mental health.
• Adult child education may be more relevant in middle-income countries due heighted reliance on family support.
• We conducted a quasi-experimental study on this topic using data in Mexico.
• Increased child schooling was associated with decreased depressive symptoms for older parents.
• Associations varied by the parent and child gender.

## 1 Introduction

Psychosocial well-being, including depression and life satisfaction, are critical endpoints for older adults' quality of life (Sivertsen et al., 2015; Şahin et al., 2019). In addition, depression may contribute to the development, management, and progression of other acute and chronic conditions (i.e., dementia, heart disease, cancer), all of which may in turn contribute to lower life satisfaction (Krishnan et al., 2002). A large and longstanding body of evidence suggests that late-life psychosocial well-being is influenced by lifecourse socio-economic status (SES), driven by a range of mechanisms including cumulative exposure to financial and occupational stressors or privileges as well as health care access (Richardson et al., 2020). Educational attainment – a core metric of lifecourse SES – is particularly important for shaping late-life psychosocial outcomes, including depression and life satisfaction (Cohen et al., 2020; Powdthavee et al., 2015). More recently, scholars have drawn on the ‘linked lives’ principle (Gilligan et al., 2018), which emphasizes the interconnectedness of resources and experiences across family generations. It entails the possibility that the education of adult children may have “upward” intergenerational effects on the psychosocial well-being of their older parents (De Neve & Kawachi, 2017). Upward intergenerational effects would have especially large implications for population health in low- and middle-income countries (LMIC), due to large recent gains in the educational attainment of younger birth cohorts (United Nations et al., 2017). Moreover, many LMIC settings are facing rapidly aging populations with poor (mental) healthcare infrastructure (Berenzon et al., 2009). Given its comorbid nature and high level of stigmatization, depression is often misdiagnosed and undertreated among older adults in Mexico and other LMICs (Mascayano et al., 2016), lending urgency around the need to understand population-level drivers of late-life psychosocial well-being.

Recent observational studies carried out across the United States, Europe, and Taiwan have shown positive associations between adult child educational attainment and psychosocial well-being among older parents (Dennison & Lee, 2021; Lee et al., 2017; Sabater & Graham, 2016; Yahirun et al., 2020). However observational studies may be biased by residual confounding. For example, unmeasured or poorly measured aspects of older adults' lifecourse socio-economic and health-related experiences may influence both adult child educational attainment as well as late-life health. To overcome this limitation a nascent body of research has leveraged changes to compulsory schooling laws as instrumental variables (Ma, 2019; Torres et al., 2022). The intuition behind these studies is that a mandatory increase in minimum school leaving age should be independent of factors that typically confound the relationship between adult child education and parents' health. These studies have found positive associations between increased schooling among adult children and older parents' quality-of-life scores and depressive symptoms in Europe (Torres et al., 2022) and with older parents’ life satisfaction (but not depressive symptoms) in China (Ma, 2019). However, studies that reflect a broader global context are critically needed.

### 1.1 Potential mechanisms linking adult child education to parents' psychosocial well-being

Adult children with higher education may influence parents' psychosocial outcomes by yielding more socioeconomic resources through financial cash transfers (Lee, 2018; Ma, 2019) and increased access to and quality of healthcare services (Lee et al., 2017). The economic security offered by more highly educated offspring who may enjoy, on average, higher-status occupations and larger incomes, may be linked to improved psychological well-being by reducing experiences of family-level financial strain. Children with higher education may also be equipped with greater informational resources that may contribute to parents' health-promoting behaviors and better opportunities for the prevention and treatment of chronic health conditions (Friedman & Mare, 2014; Gutierrez et al., 2023; Torssander, 2013; Zimmer et al., 2002, 2007), all of which may be protective of psychosocial well-being in late life (DeJean et al., 2013; Ohrnberger, Fichera, & Sutton, 2017). Adult child education is of particular importance in Mexico since most older adults don’t have a formal pension, often rely on conditional cash transfers, and are either uninsured or utilize government-based health insurance which provides limited access to mental healthcare services (Aguila et al., 2012; Espinoza & Wong, 2004). Higher education among adult children may also be linked to higher levels of social engagement with older parents (Lee et al., 2017) or an improved sense of social standing and parental pride, which may also have a positive impact on overall well-being, including reduced depressive symptoms and increased life satisfaction (Lee, 2018).

### 1.2 Different global contexts may shape the relationship with adult child education

Despite the foundational prior research on this topic, there is a critical need for research across global contexts where associations between adult child educational attainment and older parents' psychosocial well-being may vary. For example, Mexico has a limited welfare system and health infrastructure includes few formal long-term services or supports for vulnerable older adults. In this context, adult child SES may be more influential for the well-being of older adults as compared to settings like Europe, where universal health care is typical. Similar to China, but unlike Europe, intergenerational financial support is very high in Mexico and late-life care is largely expected to come from family members (Gomes, 2007; Lei, 2013). In contrast to Europe or China, Mexico has a long-standing history of substantial international emigration, which remains an ongoing phenomenon (Passel, Cohn, & Gonzalez-Barrera, 2012). This unique context may be differentially influenced by the CSL reform. Given the varying push and pull mechanisms, migrants may exhibit a range of educational backgrounds (Villarreal, 2014), prompting exploration into whether the impact of adult child schooling on parents' psychosocial well-being is attenuated in a country experiencing significant emigration of working-age adults. Increased adult child schooling may also be more strongly associated with older parents' mental health in Mexico which has experienced dramatic increases in average educational attainment among younger birth cohorts (KC et al., 2010; Urbina, 2018). In particular, the majority of the older Mexican population has less than primary schooling (Instituto Nacional de Estadística Geografíae Informática, 2005). It could be that increases in adult child educational attainment may be particularly impactful for older parents who themselves have relatively low levels of education attainment.

### 1.3 The present study

To address these gaps in prior literature, we aimed to understand how the psychosocial well-being of older parents in Mexico is impacted by increased years of schooling completed by their offspring. We used adult child exposure to the 1993 Constitutional Amendment reform, which elevated the mandatory minimum level of schooling from six to nine years, as an instrumental variable (see eAppendix 1 for contextual details). Drawing on fundamental cause theory (Phelan & Link, 2005), and the prior evidence supporting that adult child SES can confer various benefits to parents (Lee, 2018; Lee et al., 2017; Ma, 2019), we hypothesized that increases in the length of children’s schooling would be associated with improved parental psychosocial well-being in Mexico.

We further expected that there may be differences in associations by the gender of the older parent (Belló et al., 2005) and their child (Ma et al., 2021; Torssander, 2013; Yang et al., 2016). While neither of the two prior quasi-experimental studies on this topic identified heterogeneity by gender, select studies focused on other health outcomes (e.g. cognitive outcomes) have found evidence of greater returns to adult child educational attainment for older mothers (Ma et al., 2021). A smaller number of studies have also found differences in associations by adult child gender, which may be driven by gendered differences in family expectations related to economic transfers and/or time and caregiving (Friedman & Mare, 2014; Liu et al., 2022; Yahirun et al., 2016).

Finally, most studies on this topic have not considered heterogeneity based on other characteristics, including parents' own socioeconomic status and their child’s co-residence status (Courtin & Avendano, 2016). Drawing on resource substitution theory (Ross & Mirowsky, 2011), we hypothesize that parents with lower socioeconomic status are more likely to benefit more from the resources of their children, to help mitigate a critical gap in their financial and health needs. Conversely, since children with higher education may be less likely to reside in the same household or live in the same city as their parents, the impacts of increased adult child schooling could vary by contemporaneous co-residence status.

## 2 Methods

### 2.1 Data source

Data came from the 2012 wave of the Mexican Health and Aging Study (MHAS) a national cohort study of Mexican adults aged 50 or older and their spouse/partner regardless of age. The MHAS is unique in that it provides rich data on health outcomes, and it also includes information on the educational attainment of all adult children unconditional on coresidence, unlike other population-based data sources (e.g., census). Additional cohort details have been provided elsewhere (Wong et al., 2017). Briefly, the first wave of participants were interviewed in 2001 with follow-ups completed in 2003, 2012, 2015, and 2018. The response rates for these waves were 91.8%, 93.3%, 88.1%, 88.3%, and 84.7%, respectively (MHAS, 2020; Wong et al., 2017). A new sample of individuals born from 1952 to 1962 (n = 5896) were added in 2012.

Our analysis focused on both ongoing and new MHAS respondents 50+ surveyed in 2012 (n = 13,654). We selected the 2012 wave as it was comprised of participants covering birth cohorts from 1895 to 1962, which allowed us to select an analytic sample that was relatively balanced in terms of the distribution of exposure to increased adult child educational attainment via the 1993 compulsory schooling law (CSL). Given the birth cohorts of their adult children, participants in the 2001 wave were almost entirely unexposed while participants in the 2018 refresher wave were almost entirely exposed based on our specification of the instrumental variable. We excluded respondents who did not have at least one living child (n = 577). We further limited our sample to respondents whose oldest child was born in one of the 10 birth cohorts affected by the CSL or one of the 10 birth cohorts who just missed benefitting from the CSL (i.e. 1969-1989) (n = 5372 excluded), as described below. Finally, we excluded those missing information on psychosocial well-being (n = 215), adult child education (n = 36), or covariates (n = 268). Our final analytic sample consisted of 7186 older adults aged 50 and older in 2012 (eFigure 1).

### 2.2 Psychosocial well-being measures

Past-week depressive symptoms were assessed at each wave using a nine-item modified Center for Epidemiologic Studies Depression Scale (Radloff, 1977). Items were reported as binary “yes/no” responses and summary counts ranged from 0 to 9 with higher values indicating more depressive symptoms.

Life satisfaction was assessed using an adapted version of the Satisfaction with Life Scale (SWLS) (López-Ortega et al., 2016). The adapted questionnaire used the same 5 items as the original scale, and each item is scored from 1 to 3. The possible range of scores of the adapted SWLS scale included in the MHAS is from 5 (high satisfaction) to 15 (low satisfaction). Further details on measures are included in eAppendix 2.

### 2.3 Adult child educational attainment

At each wave, MHAS respondents provided information on the total years of education of each of their children who were aged ≥12 years. The primary exposure was defined as the years of education attained by the oldest child. Given that the education of the oldest child may impact the schooling of subsequent siblings (e.g., via differential resources devoted by parents, younger children following educational trajectories of older siblings as role models), this specification avoids potential interference with birth order (Bouchey et al., 2010; McHale et al., 2012). This would isolate the effect of reform on increases in the adult child’s education without the potential influence of differential parenting expectations and resources devoted to their children. We also considered the educational attainment of the oldest daughters and oldest sons separately, given prior evidence of gender heterogeneity and varying mechanisms on mental health outcomes (Lee et al., 2017). In sensitivity analyses described further below, we also considered a) the educational attainment of respondents' highest educated child and b) the educational attainment of all of their adult children.

### 2.4 Instrumental variable

Lower-secondary school completion became a national requirement in Mexico after educational policy reforms in 1993, effectively raising the minimum mandatory years of schooling from 6 to 9 years (Reforma 2019 a los artículos, 2019). This policy change implies that children born in 1979 or later would have been exposed to the reform changes. We use the exogenous variation in education induced by the reform to identify the effects of children’s education on parents' psychosocial well-being. We limited the sample to respondents whose oldest children were in birth cohorts within ten years on each side of the cut-off for eligibility to minimize any residual confounding due to respondent age (which is likely correlated with oldest child age) or other co-occurring historical events or social changes. The binary instrumental variable was an indicator of whether the oldest child of the respondent was born in the first 10 birth cohorts affected by the CSL (and therefore exposed to the educational policy reform) or born in the 10 birth cohorts that would have just missed being impacted by the same law (i.e. the control group).

### 2.5 Covariates

We controlled for respondents' demographic characteristics (e.g., age, gender, marital status) and mid-life SES measures (e.g., educational attainment, indigenous dialect, parental education, childhood disadvantage score, primary lifetime occupation) as well as demographic characteristics of respondents’ spouses, if relevant. Further details on the confounder variables are included in the eAppendix 2.

### 2.6 Statistical analyses

We used two-stage least squares (2SLS) estimation to evaluate the effects of increased schooling among the oldest children on parents’ scores on each of the psychosocial measures described above. We compared these estimates to those generated via conventional Ordinary Least Squares (OLS) adjusted for all covariates.

We invoked conventional assumptions for instrumental variables (IV) analysis, including relevance (i.e., the instrument (change in CSL) has a causal effect on the exposure (adult child education)), exclusion restriction (i.e., the CSL is only associated with parental psychosocial well-being through increases in adult child schooling), independence (i.e., the CSL and parental psychosocial well-being have no shared common causes after accounting for measured confounders), and monotonicity (i.e., no adult children achieved below the minimum levels of schooling because of the CSL or that were no ‘defiers’ of the CSL) (Hernan & Robins, 2023; Labrecque & Swanson, 2018). Further assumption details available in eAppendix 3.

For the 2SLS approach, we generated first-stage estimates using a linear model that regressed the years of schooling completed by the oldest child on the binary instrumental variable and covariates. The first-stage model is defined as:

(Eq. 1) $${Years\;of\;Schooling}_{i\;}=\propto_{0\;}+\propto_{1\;}{CSL}_{i\;}+\propto_{2\;}V_{i\;}+\varepsilon_{ih}$$

where ${Years\;of\;Schooling}_{i\;}$ indicates the years of schooling reported for the index child for respondent, i; ${CSL}_{i\;}$ is a birth cohort-based variable indicating whether the respondents' index child was in the first 10 birth cohorts exposed to the compulsory schooling law vs. those in the 10 birth cohorts who just missed benefitting from the change; $V_{i\;}$ is a vector of individual-level covariates; and $\varepsilon_{ih}$ is the error term, clustered at the household level *h* to account for multiple interviews per household (i.e., spouses).

The second-stage model regressed respondents’ psychosocial variable (either depressive symptoms or life satisfaction score) on the predicted value of educational attainment for their oldest educated child, generated with the first-stage regression, and the same set of covariates as the first-stage model. In the models for the overall sample, we clustered the standard errors at the household level to account for spousal respondents living in the same dwelling. The second-stage model is defined as:

(Eq. 2) $${Psychosocial\;Outcome}_{i\;}=\beta_{0\;}+\beta_{1\;}{\hat{Years\;of\;Schooling}}_{i}+\beta_{2\;}V_{i}+\varepsilon_{ih}$$

where the outcome is the score of the given psychosocial measure for each respondent, i; ${\hat{Years\;of\;Schooling}}_{i}$ corresponds to the predicted value of years of schooling attained by the respondents' oldest children generated from (Eq. (1)); $V_{i}$ is the same vector of baseline covariates that were included in the first-stage model; and $\varepsilon_{ih}$ is the error term, clustered at the household level. We also estimated models stratified by the parent (respondents') and child’s gender.

To understand the impacts of the reform itself, we additionally generated reduced form estimates where the respondents’ psychosocial variable (either depressive symptoms or life satisfaction score) was regressed directly on the CSL birth-cohort indicator:

(Eq. 3) $${Psychosocial\;Outcome}_{i\;}=\propto_{0\;}+\propto_{1\;}{CSL}_{i\;}+\propto_{2\;}V_{i\;}+\varepsilon_{ih}$$

#### 2.6.1 Evaluating heterogeneity and sensitivity analyses

In supplementary models, we explored the potential for heterogenous effects of the reform across several socio-demographic characteristics. We tested whether the effects differed based on whether the oldest child was co-residing with their parents (respondents), the parent’s (respondent’s) educational attainment (0–5 vs. 6+ years), and whether the parent (respondent) was currently living in an urban area.

We also conducted several sensitivity analyses. First, we used the highest-educated child as the index child, following prior literature (Ma et al., 2021). When multiple children had the same highest level of schooling, we selected the oldest highest educated child as the index child. As an alternative instrument specification, we used the average years of schooling required for all children (25+) to capture the impacts of increased schooling across all of respondents’ adult children. Additionally, we evaluated results across a series of different birth cohort bounds (5 and 10 years) to investigate the effect varying sample sizes have on our estimates. We excluded the first two birth cohorts impacted by the reform to account for potential delays in policy implementation. Finally, we evaluated an alternate specification of depressive symptomatology. Analyses were performed using Stata 17.0 (StataCorp, College Station, TX).

## 3 Results

### 3.1 Descriptive characteristics

Respondents (older parents) were an average of 60.1 years old (standard deviation (SD), 6.5) (range, 50–110), a majority were women (54.9%), with a mean depressive symptoms of 3.2 (±2.6 SD) and life satisfaction score of 7.0 (±2.3 SD) (Table 1). Respondents reported that they had achieved an average of 6.7 years of education (±4.8), but that their oldest children had completed a mean of 11.2 (SD, 4.4) years of schooling. Respondents whose oldest adult child were in the first 10 birth cohorts to benefit from the CSL (n = 2833) were more likely to be younger, male, and have greater own educational attainment as compared to respondents whose oldest child was in the 10 birth cohorts that just missed benefitting from the reform (n = 4353).

**Table 1:** Baseline descriptive characteristics of older Mexican adults 50+ years whose oldest child was in the first 10 birth cohorts to benefit from or 10 birth cohorts to just miss benefiting from a change in compulsory schooling laws, Mexican Health and Aging Study, 2012

	Overall (n = 7186)			Not impacted by the CSL (n = 4353)			Impacted by the CSL (n = 2833)			*P*
	No. (%)	Mean	SD	No. (%)	Mean	SD	No. (%)	Mean	SD	
*Respondent demographic characteristics*										
Age, years		60.14	6.48		62.55	5.93		56.44	5.46	<0.001
Gender										<0.001
Women	3948 (54.94)			2502 (57.48)			1446 (51.04)			
Men	3238 (45.06)			1851 (42.52)			1387 (48.96)			
Marital status										<0.001
Married	5774 (80.35)			3446 (79.16)			2328 (82.17)			
Widowed	689 (9.59)			541 (12.43)			148 (5.22)			
Divorced, separated, never married	723 (10.06)			366 (8.41)			357 (12.60)			
Spouse age, years [a]		59.34	7.33		62.23	6.40		54.96	6.42	<0.001
Total number of children		4.25	2.27		4.70	2.38		3.55	1.89	<0.001
*Respondent and family life-course socioeconomic status*										
Respondent years of education		6.66	4.75		5.89	4.46		7.85	4.92	<0.001
Speaks an indigenous dialect	525 (7.31)			314 (7.21)			211 (7.45)			0.71
Spouse years of education		6.63	4.70		5.91	4.47		7.71	4.82	<0.001
Highest level of parental education										<0.001
None	2207 (30.70)			1445 (33.20)			762 (26.90)			
Some elementary	2800 (38.96)			1663 (38.20)			1137 (40.13)			
Completed elementary	1020 (14.19)			558 (12.82)			462 (16.31)			
More than elementary	666 (9.27)			346 (7.95)			320 (11.30)			
Missing for both parents	493 (6.86)			341 (7.95)			152 (5.37)			
Childhood disadvantage score (0–7) [b]		1.89	1.54		1.93	1.53		1.83	1.54	0.007
Respondent lifetime primary occupation										<0.001
Never worked for pay	2406 (33.50)			1813 (41.66)			593 (20.95)			
White collar	1084 (15.09)			448 (10.29)			636 (22.47)			
Blue collar	3046 (42.41)			1720 (39.52)			1326 (46.84)			
Agricultural	647 (9.01)			371 (8.52)			276 (9.75)			
Spousal lifetime primary occupation										<0.001
Never worked for pay	1916 (27.43)			1370 (32.14)			546 (20.06)			
White collar	793 (11.35)			349 (8.19)			444 (16.31)			
Blue collar	2332 (33.39)			1310 (30.73)			1022 (37.55)			
Agricultural	532 (7.62)			327 (7.67)			205 (7.53)			
*Adult child educational attainment*										
Years of educational attainment, oldest child		11.24	4.37		10.79	4.35		11.92	4.31	<0.001
Years of educational attainment, oldest daughter [c]		10.95	4.33		10.39	4.31		11.70	4.25	<0.001
Years of educational attainment, oldest son [d]		10.80	4.33		10.33	4.36		11.41	4.22	<0.001
*Psychosocial outcomes*										
Depressive symptoms. CES-D (range: 0–9) [e]		3.17	2.58		3.32	2.64		2.95	2.48	<0.001
Life satisfaction score, SWLS (range: 5–15) [f]		6.95	2.31		6.92	2.31		7.00	2.33	0.13

Abbreviations: CES-D, Center for Epidemiologic Studies Depression Scale; CSL, compulsory schooling law; SD, standard deviation; SWLS, Satisfaction with Life Scale.

a Limited to those who were married at baseline.

b Disadvantage score generated from seven items. Respondents were asked if before the age of 10, they experienced any of the following: did not have access to sanitation facilities, generally went to bed hungry, did not regularly wear shoes, self/siblings had to drop out of school to support family, self/family slept in the kitchen, family received financial support, had a serious health problem that affected their normal activities.

c Limited to n = 6725 with at least 1 adult daughter.

d Limited to n = 6707 with at least 1 adult son.

e The CES-D scale is a measure of past-week depressive symptoms, with scores ranging from 0 (no symptoms) to 9.

f Items were coded such that higher scores reflected lower quality of life, with scores ranging from 5 to 15.

### 3.2 Reduced form estimates

eFigure 2 displays the mean depressive symptoms and life satisfaction scores by the child’s birth cohort. A child’s exposure to the reform was associated with fewer depressive symptoms (β = −0.16; 95% CI: −0.30, −0.01) among older parents; associations with life satisfaction were null (β = 0.01; 95% CI: −0.14, 0.15) (eTable 1). These results can be interpreted as the ‘intent-to-treat’ estimates of the schooling reform, given that it conflates the effect of the child being exposed to the CSL among parents whose children increased their schooling because of the policy change and those who did not.

### 3.3 First-stage results

eFigure 3 displays the average educational attainment of oldest adult children by the child’s birth cohort. Exposure to the CSL reform was associated with an increase in average educational attainment by 0.62 years among oldest children (95% CI: 0.37, 0.86), 0.84 years for oldest daughter (95% CI: 0.60, 1.09), and 0.48 years for oldest sons (95% CI: 0.23, 0.74) (Table 2). The Kleibergen-Paap Wald F-statistics for the first-stage were 24.17, 45.80, and 14.30. All subsequent second-stage results had a first-stage F-statistic above the conventional cut-off of 10.

**Table 2:** Beta coefficients and 95% confidence intervals for first-stage linear regression of years of schooling completed by the oldest child on the birth cohort-based instrument and Kleibergen-Paap Wald F-tests.

	β	[95% CI]	F
Years of schooling, oldest child	0.62	0.37, 0.86	24.17
Years of schooling, oldest daughter	0.84	0.60, 1.09	45.80
Years of schooling, oldest son	0.48	0.23, 0.74	14.30
Years of schooling, highest educated child	0.41	0.21, 0.61	15.99

The CSL reform primarily increased adult child schooling from a 9-year minimum threshold (resulting in a 3.7% increased probability) to a minimum of 12 years (with a 7.6% increased probability). The most significant impact of the CSL was observed in raising the probability of adult children having at least 10 years of schooling by 9.5%, compared to those not exposed to the reform (eTable 2).

### 3.4 Oldest child’s educational attainment and parents' psychosocial outcomes

The OLS regression estimates suggested that each additional year of schooling for an oldest child was associated with fewer depressive symptoms (β = −0.04, 95% CI: −0.05, −0.02) and greater life satisfaction (β = −0.04, 95% CI: −0.05, −0.02; Table 3 Panel A) among parents.

**Table 3:** Comparison of beta coefficients and 95% confidence intervals from ordinary least squares and two-stage least squares regression evaluating the association between oldest child’s educational attainment and older parents' psychosocial outcomes in Mexico, overall and by gender.

Panel A: Overall				
	Oldest Child (n = 7186)			
	OLS		2SLS	
	β	95% CI	β	95% CI
Depressive symptoms (0–9)	−0.04	(−0.05, −0.02)	−0.25	(−0.51, 0.00)
Life satisfaction score (5–15)^a^	−0.04	(−0.05, −0.02)	0.01	(−0.22, 0.25)

Panel B: Parent’s Gender								
	Fathers (n = 3238)				Mothers (n = 3948)			
	OLS		2SLS		OLS		2SLS	
	β	95% CI	β	95% CI	β	95% CI	β	95% CI
Depressive symptoms (0–9)	−0.03	(−0.06, −0.01)	−0.18	(−0.63, 0.26)	−0.04	(−0.06, −0.02)	−0.27	(−0.56, 0.01)
Life satisfaction score (5–15)^a^	−0.03	(−0.05, −0.01)	0.28	(−0.18, 0.76)	−0.04	(−0.06, −0.02)	−0.08	(−0.34, 0.17)

Panel C: Adult Child’s Gender								
	Oldest Daughter (n = 6725)				Oldest Son (n = 6707)			
	OLS		2SLS		OLS		2SLS	
	β	95% CI	β	95% CI	β	95% CI	β	95% CI
Depressive symptoms (0–9)	−0.03	(−0.04, −0.01)	−0.05	(−0.23, 0.13)	−0.05	(−0.06, −0.03)	−0.37	(−0.73, −0.02)
Life satisfaction score (5–15) a	−0.03	(−0.04, −0.01)	0.09	(−0.08, 0.25)	−0.05	(−0.06, −0.03)	0.19	(−0.13, 0.51)

a Items were coded such that higher scores reflected lower quality of life.

**Fig. 1:**
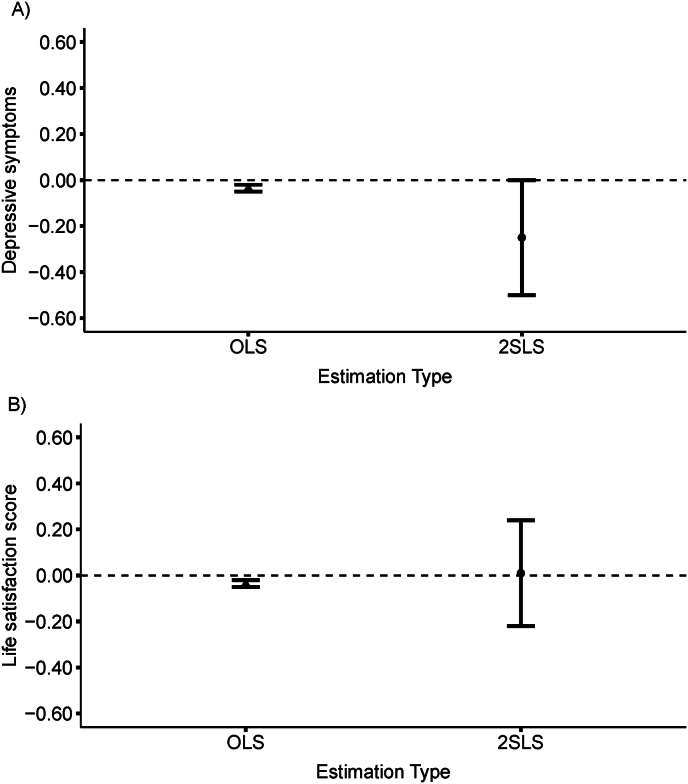
Two-stage least squares and ordinary least squares (OLS) regression estimates, oldest child’s schooling duration, and older parents' psychosocial well-being in the Mexican Health and Aging Study, 2012. A) depressive symptoms; b) life satisfaction score. Depressive symptoms were coded such that higher scores reflected greater depressive symptomatology, with scores ranging from 0 to 9. Life satisfaction was coded such that higher scores reflected lower quality of life, with scores ranging from 5 to 15.

In the IV models, each 1-year increase in the schooling duration of the oldest adult child was associated with fewer past-week depressive symptoms for older parents (β = −0.25; 95% CI: −0.51, 0.00) but associations with life satisfaction were null (β = 0.01; 95% CI: −0.22, 0.25; Fig. 1).

IV models stratified by parent gender (Table 3 Panel B) suggested that the association between increased adult child schooling and fewer depressive symptoms was driven by older mothers (β = −0.27, 95% CI: −0.56, 0.01), instead of fathers (β = −0.18, 95% CI: −0.63, 0.26), although 95% CIs crossed the null in both cases and overlapped with one another. There were no meaningful differences in the association with life satisfaction scores by parent gender.

### 3.5 Oldest daughter’s and son’s educational attainment and parents' psychosocial outcomes

**Fig. 2:**
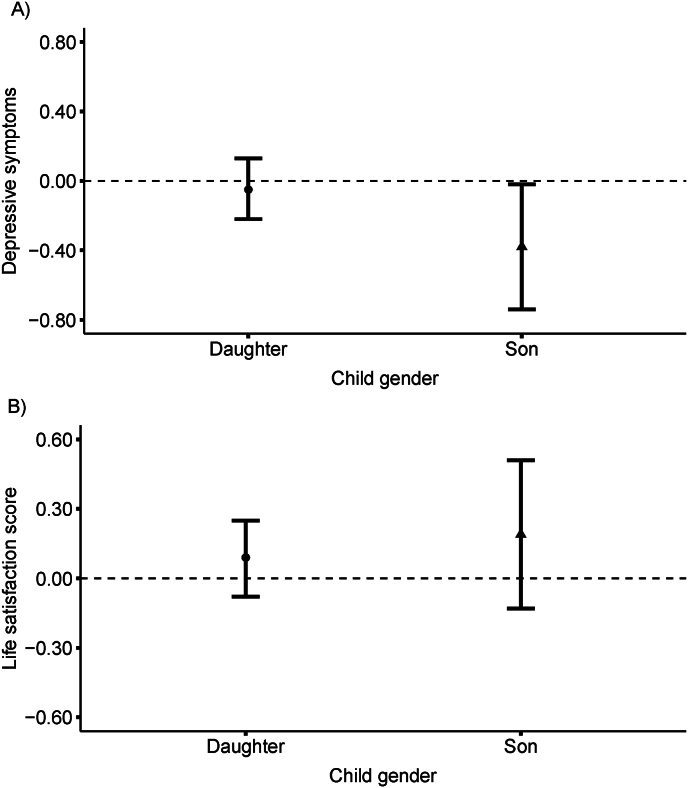
Two-stage least squares (2SLS) estimates of the association between oldest child’s schooling duration and older parents' psychosocial well-being, by child gender in the Mexican Health and Aging Study, 2012. A) depressive symptoms; b) life satisfaction score. Depressive symptoms were coded such that higher scores reflected greater depressive symptomatology, with scores ranging from 0 to 9. Life satisfaction was coded such that higher scores reflected lower quality of life, with scores ranging from 5 to 15.

OLS estimates indicated that each additional year of schooling for the oldest daughters (β = −0.03, 95% CI: −0.04, −0.01) and oldest sons (β = −0.05, 95% CI: −0.06, −0.03) were similarly associated with fewer depressive symptoms and greater life satisfaction (β = −0.03, 95% CI: −0.04, −0.01 for daughters and β = −0.05, 95% CI: −0.06, −0.03 for sons) among older parents (Table 3 Panel C; Fig. 2).

In the IV models, we observed differences in the magnitude of association between oldest daughter’s (β = −0.05, 95% CI: −0.23, 0.13) and oldest son’s (β = −0.37; 95% CI: −0.73, −0.02) education with parents' depressive symptoms. Associations with life satisfaction continued to be null.

The association between increased schooling among oldest daughters and parents’ depressive symptoms did not appear to vary by parental gender (eTable 3). Increased schooling among oldest sons was associated with fewer depressive symptoms (β = −0.42, 95% CI: −0.82, −0.02) among older mothers, but associations were null for oldest fathers (β = −0.29, 95% CI: −0.88, 0.30) (eTable 4).

### 3.6 Evaluating heterogeneity

We aimed to further evaluate heterogeneity by adult child-respondent coresidence status, respondents’ own educational attainment, and urban locality in analyses stratified by these characteristics. However, first-stage estimates suggested that the CSL-based instrument was weak for respondents who co-resided with the index child, for respondents with less than a primary school education, and for those residing in rural areas. We therefore proceeded with second-stage analyses only for respondents who did not co-reside with the index child, respondents with at least a primary school education, and those residing in urban areas (eTables 5–7). Overall, estimates for these subgroups were similar to those generated in our primary analyses (albeit much less precise), although estimates for respondents who had completed at least a primary education were of somewhat smaller magnitude compared to our main results (β = −0.16, 95% CI: −0.37, 0.06 for depressive symptoms; β = −0.02, 95% CI: −0.25, 0.20 for life satisfaction, eTable 6).

### 3.7 Sensitivity analyses

In sensitivity analyses, both OLS and first-stage point estimates were of smaller magnitude when we used the highest educated (vs. oldest) child as the index child, but confidence intervals overlapped substantially with our primary estimates (eTable 8). The second-stage estimates for both depressive symptoms and life satisfaction were generally similar compared to our primary analyses. We found no evidence of heterogeneity by parent gender when instead focusing on the highest educated child (eTable 9).

The first-stage estimates using an alternative instrument that averaged the compulsory schooling required for all adult children (25+) in the family (eTable 10) were smaller than those generated in our analyses focusing on the oldest child (β = 0.13 years of schooling; 95% CI: 0.05, 0.22, partial F-statistic: 9.44). The second-stage estimates of the association between each additional year of schooling averaged across all adult children and older parents’ depressive symptoms (β = −0.21; 95% CI: −0.69, 0.26) were of nearly the same size as those reported in our primary analyses, although were highly imprecise. We observed an association between each additional year of schooling averaged across adult children and higher life satisfaction scores among older parents (β = 0.48; 95% CI: −0.07, 1.04), in contrast to our primary results, although the 95% confidence interval crossed the null.

When we used 5-year birth cohort bounds to achieve a better balance between the treated and control groups, first-stage estimates were weaker (eTables 11-12); the second-stage estimates for depressive symptoms were of larger magnitude but less precise. Conversely, when we used 15-year bounds we observed findings consistent findings with our primary analyses (eTables 11 and 12). We found consistent but less precise estimates when we excluded the first two birth cohorts impacted by the reform due to the smaller sample size (eTable 13). Results were consistent when using a binary measure of elevated depressive symptomatology (eTable 14).

## 4 Discussion

In this population-based quasi-experimental analysis, we found that increased schooling among adult children was associated with fewer depressive symptoms for older adults in Mexico, although associations with life satisfaction were null. As expected, our reduced form estimates were attenuated from those using 2SLS estimation due to non-compliance. We additionally found modest evidence of gender differences in the returns to adult child schooling whereby the magnitude of associations were greater for older mothers as compared to older fathers. There was also evidence that the association between increased schooling among adult sons and older parents’ depressive symptoms was of greater magnitude than that observed for adult daughters. These findings add critical evidence to the nascent body of research on the upstream influence of adult child socio-economic status on the outcomes of older parents. Most of the research on this topic that has focused on psychosocial outcomes has been observational, which may be subject to residual confounding.

To place these findings in context, we benchmarked our estimates against estimates of an association between basic activities of daily living (ADLs; e.g., difficulty with eating, bathing, and getting out of bed) – a well-established predictor of depression in older adults (Ormel et al., 2002, pp. P338–P347) – and depressive symptoms (Gutierrez et al., 2022). We calculated that each additional ADL limitation (range 0–6) was associated with an average increase of 0.45 in depressive symptoms (95% CI: 0.41, 0.49), controlling for sociodemographic characteristics and self-rated health. This suggests that the magnitude of the association observed between each one-year increase in adult child schooling and parental depressive symptoms was approximately equivalent to 0.55 fewer ADL limitations. Given that the change in CSL that we focused on – and many other changes in CSLs across global settings – have intended to raise average educational attainment by as many as 3 years, this means that such policies may have substantial impacts on late-life depression, or nearly the equivalent of removing 1.65 ADL limitations.

Our findings are consistent with observational studies showing protective associations between adult child education and parents' depressive symptoms conducted in other global settings, including in Taiwan and the United States (Lee et al., 2017; Yahirun et al., 2020). A direct comparison to these prior studies is hampered by the fact that some of these prior studies adjusted for potential mediators of the association of interest, such as older parents' income and wealth (Sabater & Graham, 2016; Yahirun et al., 2020). However, our findings diverge to some extent from the two other quasi-experimental studies on this topic (Ma, 2019; Torres et al., 2022). Contrary to our findings, Ma found a null quasi-experimental association between increased adult child schooling and older parents’ past-week depressive symptoms, but a protective association with life satisfaction in a study of older Chinese adults (Ma, 2019). However, in a study of older European adults, Torres et al., found that increased adult child schooling was associated with both higher quality of life scores and fewer past-month depressive symptoms (Torres et al., 2022). Both of these prior studies leveraged birth cohort-based measures of exposure to changes in compulsory schooling laws as instrumental variables.

Differences between our findings and those reported by prior quasi-experimental studies may be driven by contextual and/or methodological factors. One potential methodological difference concerns the selection of the index child to consider as exposed or unexposed to CSLs (Peng et al., 2019). Ma selected the highest educated child, whereas our study and Torres et al. selected the oldest child. Similar to Torres et al., our sensitivity analyses using the highest educated child resulted in smaller and less precise estimates. We additionally considered all adult children and found similar, but largely imprecise estimates for depressive symptoms. Beyond these methodological differences, there are a number of potential contextual drivers of different findings, including differences in the CSLs themselves. We focused on a CSL in Mexico that increased education by 3 years, but the varied CSLs passed in European countries increased schooling from 1 to 4 years. In addition, the mandatory minimum levels of schooling prior to the European reforms considered by Torres et al. were generally higher (e.g. secondary schooling or higher), whereas prior to the 1993 CSL, minimum schooling in Mexico ended at a primary education. In China – the location of the only other quasi-experimental study on this topic – the CSL had similar parameters as the 1993 CSL in Mexico (i.e. from 6 to 9 years), although the impacts appear to have been concentrated among rural Chinese municipalities. In contrast, we found that we could not even estimate second-stage results for respondents in non-urban communities due to the very weak first-stage evidence, pointing to the potential concentration of benefits for urban residents in the Mexican context.

There may be other contextual drivers of differences between our findings and those reported by other studies. For example, our findings of no quasi-experimental association between adult child education and older parents' life satisfaction scores could suggest that other factors, such as social support, health status, or one’s own economic conditions, may play a more prominent role in shaping parents' overall life satisfaction in the Mexican context. However, prior work in the MHAS has found life satisfaction to be correlated with depressive symptoms. It could also be that null findings with life satisfaction are driven by countervailing adverse impacts of higher adult child educational attainment. For example, more highly educated children may be more likely to move away from their older parents to pursue job prospects that are in better alignment with their education. Given the strong family ties and filial responsibility placed on children in Mexico, increases in education could also adversely impact life satisfaction if it is associated with a reduced probability of co-residence or residence nearby. Our first-stage results by child residence status help support this since respondents whose child did not co-reside had stronger estimates. However, more work is needed to understand the nuanced family dynamics that may modify the health impacts of increased adult child educational attainment.

We found evidence of differences in the magnitude of the association between adult child schooling and parents' depressive symptoms by parent and child gender; however, the confidence intervals overlapped throughout. Drawing on the theory of resource substitution (Ross & Mirowsky, 2011), scholars have theorized that the greater health returns to adult child education for older mothers might be because women are more likely to have lower SES than their male counterparts and are more likely to be widowed. This may be more pronounced in Mexico, given that currently less than half of women participate in the labor market and the persistent gender gap in educational attainment. In contrast, prior quasi-experimental studies either found no differences according to parent gender (Torres et al., 2022) or didn’t explore gender heterogeneity (Ma, 2019). An observational study based in Taiwan found no significant gender differences in the association between adult child educational attainment and older parents' depressive symptoms, despite some overlapping contextual characteristics to Mexico (i.e., fewer educational and employment opportunities for older generations of women) (Lee et al., 2017). These differences may be driven by the smaller sample sizes in the Taiwanese study or methodological differences.

Our findings also present suggestive evidence that the association between increased adult child schooling and fewer depressive symptoms may be driven by increased schooling for sons vs. daughters. This is contrary to our expectations given that daughters tend to have greater responsibility to support and care for their older parents in Mexico (Ybañez Zepeda et al., 2005). One possible driver of this difference may be that in the context of Mexico, increased levels of schooling for children may result in higher labor force participation rates among sons compared to daughters (Gurría, 2020; Novta & Wong, 2017). Therefore, sons are more likely to be employed and earn income. Consequently, this could lead to greater financial transfers from sons to their older parents, which could help alleviate financial strain for the parents and may have greater downstream implications for parents' depressive symptoms. Another factor that may augment the impact of varied labor market returns is the higher likelihood of men emigrating in search of economic opportunities. With the ability to send remittances, potentially of greater value due to higher wages in the US, this may result in improved material circumstances for their older parents. While this may lead to the loss of emotional and instrumental support for older parents (Torres et al., 2018), our findings suggest that the association between increased son’s schooling and improved parental psychosocial well-being may be primarily economic in nature. However, our sample size is too small to formally test whether there is heterogeneity by immigrant status and gender of the index child. We posit that these differences could partly depend on gendered trends in education, returns to education, labor-market participation, migration patterns, and familial caregiving in Mexico and these analyses shed some light on these gender nuances. More research is needed to better understand the underlying intersecting mechanisms driving this gender differential.

We expanded on prior studies by considering the heterogeneous returns to parents' own socio-economic status. First-stage results were strongest among higher SES respondents (i.e. those who at least completed primary school and lived in an urban locality). This may be driven by the fact that those with more resources and living in urban (vs. rural areas) may have been able to afford to keep their kids in school longer in Mexico (i.e. complying with the compulsory schooling law). For those with lower own educational attainment and living in rural areas, limited accessibility of schools and other economic limitations may have contributed to smaller first-stage coefficients. Although no prior studies on this topic have reported on heterogeneity in the first-stage, we expect that in higher-income country settings, stronger first-stage estimates might be observed for parents with lower own SES, given that those with higher SES may have already been able to support greater than minimum schooling prior to the passage of the CSLs. These heterogeneous first-stage results further emphasize the potential cross-national differences surrounding relationships between intergenerational educational attainment and mental health. Our second-stage estimates were underpowered to assess whether the SES of respondents modified the association between adult child schooling and parents' well-being. Future studies should continue to assess heterogeneity in larger samples to investigate whether there are subgroup differences in both the reform uptake and its impacts on parents’ health, as this would suggest the need to tailor future reforms.

### 4.1 Limitations

Our analyses have limitations. First, estimates generated from the instrumental variables approach are interpreted as the local average treatment effect. Estimates are therefore not generalizable to families whose children’s education was not affected by the compulsory schooling law, for example, because they would have achieved higher than the minimum levels of schooling regardless of the compulsory schooling laws or because they dropped out of the minimum level of schooling even with the new laws in place. Findings might also not be generalizable to other birth cohorts or historical time periods. Second, respondents may misreport their children’s education; although, they may be more familiar with milestone levels they completed as captured by the reform increase from primary to secondary school completion. Finally, we acknowledge the potential violations of exclusion restriction and independence assumption because there may be other characteristics innate to that time period that may also be correlated with parents' psychosocial well-being. However, to reduce the influence of other potential social and historical factors we included birth cohort bounds around the instrument. Sensitivity analyses with smaller bounds yielded similar but imprecise results due to the reduced sample size. To minimize the inflation of such biases, second-stage results were only estimated for relatively strong instruments.

### 4.2 Conclusion

In conclusion, our findings contribute to the growing literature on the ‘upstream’ effects of intergenerational education and parental psychosocial well-being, with quasi-experimental evidence from Mexico. Our findings suggest that increased schooling among adult children is associated with lower depressive symptoms – but not life satisfaction - among older parents. These results suggest that social policies that intervene to improve the educational conditions of one generation may be of benefit to earlier generations (older parents), in addition to the effects such policies might have on their primary targets. Moreover, our results suggest potential gender differentials in the returns to education, with greater returns observed for increased schooling among older sons (vs. daughters) and a larger magnitude of association with depressive symptoms for older mothers. Future research should investigate mechanisms underlying the heterogeneity in this relationship in the context of varying gender-specific norms across global settings.

## Funding

The Mexican Health and Aging Study is funded by the 10.13039/100000049National Institute on Aging (R01AG018016) and the Mexico National Institute of Statistics and Geography (Aguascalientes City, Aguascalientes, Mexico). SG, JMT, and EC acknowledge funding from the National Institute on Aging (R01AG072448); EC acknowledges funding from 10.13039/100014013UK Research and Innovation (EP/Y010345/1). MMG acknowledges funding from the National Institute on Aging (RF1AG055486).

## Ethics statement

This is a secondary data analysis of publicly available data; the Institutional Review Board at the University of California San Francisco has determined it meets exempt status (#21–35802, January 02, 2022).

## CRediT authorship contribution statement

**Sirena Gutierrez:** Writing – review & editing, Writing – original draft, Methodology, Formal analysis, Data curation, Conceptualization. **Emilie Courtin:** Writing – review & editing, Writing – original draft, Methodology, Conceptualization. **M. Maria Glymour:** Writing – review & editing, Writing – original draft, Methodology, Conceptualization. **Jacqueline M. Torres:** Writing – review & editing, Writing – original draft, Methodology, Conceptualization.

## Declaration of competing interest

The authors have no relevant financial or non-financial interests to disclose.

## Data Availability

The data for this study is publicly available at http://www.mhasweb.org/.
