# Supplementary material for: Does schooling attained by adult children affect parents' psychosocial well-being in later life? Using Mexico’s 1993 compulsory schooling law as a quasi-experiment
Source: SSM Popul Health. 2024 Feb 10;25:101616. doi: 10.1016/j.ssmph.2024.101616 (PMC10905038; doi:10.1016/j.ssmph.2024.101616)
Supplement: Multimedia component 1 [file mmc1.docx]

**Supplemental Appendix for *“Does schooling attained by adult children affect parents’ psychosocial well-being in later life? Using Mexico’s 1993 compulsory schooling law as a quasi-experiment”***

eAppendix 1. Context of the 1993 Constitutional Amendment Educational Reform

eFigure 1. Flowchart depicting analytic sample selection

eAppendix 2. Description of psychosocial well-being measures and covariates

eAppendix 3. Description of the assumptions for instrumental variables analysis

eFigure 2. Graphs for the mean psychosocial well-being scores of older Mexican adults (50+), by the child’s birth cohort

eTable 1. Comparison of Beta Coefficients and 95% Confidence Intervals from Ordinary Least Squares Evaluating the Association between Exposure to the Compulsory Schooling Law Reform and Older Parents’ Psychosocial Outcomes in Mexico (n=7186)

eFigure 3. Graphs for the average educational attainment of the children of older Mexican adults (50+), by the child’s birth cohort.

eTable 2. First-Stage Linear Probability Models of the Schooling Completion Thresholds of the Oldest Child on the Birth Cohort-Based Instrument and Kleibergen-Paap Wald F-Tests (n= 7186)

eTable 3. Comparison of Beta Coefficients and 95% Confidence Intervals from Ordinary Least Squares and Two-Stage Least Squares Regressions Evaluating the Association between Oldest Daughter’s Educational Attainment and Older Parents’ Psychosocial Outcomes in Mexico, according to parent gender (n = 6725)

eTable 4. Comparison of Beta Coefficients and 95% Confidence Intervals from Ordinary Least Squares and Two-Stage Least Squares Regressions Evaluating the Association between Oldest Son's Educational Attainment and Older Parents’ Psychosocial Outcomes in Mexico, according to parent gender (n = 6707)

eTable 5. Comparison of Beta Coefficients and 95% Confidence Intervals from Two-Stage Least Squares Regressions Evaluating the Association between Oldest Adult Children's Educational Attainment and Psychosocial Outcomes for Older Parents aged 50+ in Mexico, by Coresidence Status

eTable 6. Comparison of Beta Coefficients and 95% Confidence Intervals from Two-Stage Least Squares Regressions Evaluating the Association between Oldest Adult Children's Educational Attainment and Psychosocial Outcomes for Older Parents aged 50+ in Mexico, by Parents' Educational Attainment

eTable 7. Comparison of Beta Coefficients and 95% Confidence Intervals from Two-Stage Least Squares Regressions Evaluating the Association between Oldest Adult Children's Educational Attainment and Psychosocial Outcomes for Older Parents aged 50+ in Mexico, by Urbanicity

eTable 8. The association of exposure to CSL reform on the schooling of the highest educated adult child (IV 2SLS first-stage) and the association of the highest educated child’s schooling on psychosocial outcomes (IV 2SLS second-stage and OLS) (n=8,196)

eTable 9. Comparison of Beta Coefficients and 95% Confidence Intervals from Ordinary Least Squares and Two-Stage Least Squares Regressions Evaluating the Association between the Highest Educated Adult Child’s Schooling and Older Parents’ Psychosocial Outcomes in Mexico, by parent gender

eTable 10. The association of exposure to CSL reform (instrument: average required schooling) on the average educational attainment of all children 25 years or older (IV 2SLS first-stage) and the association of average educational attainment of all children (25+) on psychosocial outcomes (IV 2SLS second-stage and OLS)

eTable 11. First-Stage 2SLS estimates and Kleibergen-Paap Wald F-Tests for Alternate Bound Specifications Birth Cohort-Based Instrumental Variable on Years of Oldest Adult Child Schooling

eTable 12. Comparison of Beta Coefficients and 95% Confidence Intervals from Two-Stage Least Squares Regressions Evaluating the Association between Oldest Child's Educational Attainment and Older Parents’ Psychosocial Outcomes in Mexico, by instrument bound type

eTable 13. Beta Coefficients and 95% Confidence Intervals from Two-Stage Least Squares Regressions Evaluating the Association between the Educational Attainment of the Oldest Child and Older Parents’ Psychosocial Outcomes in Mexico, using a 2-year wash out period

eTable 14. Comparison of Coefficients and 95% Confidence Intervals from Logit and Two-Stage Least Squares Regression Evaluating the Association between Oldest Child’s Educational Attainment and Older Parents’ Elevated Depressive Symptomatology in Mexico, Overall and by Gender

**eAppendix 1. Context of the 1993 Constitutional Amendment Educational Reform**

The presidency of Benito Juarez in 1867 laid the groundwork for secular education in Mexico and mandated primary education, although it was not truly universal.^1^ During the socialist period (1934-1946), the Ministry of Education was established, leading to an expansion in school infrastructure.^2,3^ However, it was not until the educational expansion reforms of the 11-Year Plan (1959-1969) that sustained efforts were made to expand education infrastructure across the country. This encompassed widespread construction of schools, the training of teachers, and initiatives to facilitate student attendance (e.g., free breakfasts and textbooks, standardizing the school calendar). For example, between 1958 and 1964, the federal government built 21,815 elementary classrooms, leading to an increase in enrollments from 160 to 215 students per school overall. Federal budget allocation for education rose from about 15% in 1934 to approximately 40% during this period. Nevertheless, gender, regional, and class inequities in educational attainment persisted.^2^

Leading up to Mexico’s 1993 Constitutional Amendment, educational progress in the country had stagnated due to limited reforms in the eighties caused by economic instability. Most of the gains in educational attainment were in primary schooling attainment, such that enrollment rate surpassed 98% since the early 1990s.^4^ As part of Carlos Salinas presidency, in 1993, Article 3 of the Constitution was amended to make school compulsory up to grade 9.^5^ One major change in Mexico’s school system during this time was the decentralization of education, with the federal government transferring educational responsibilities to state and local authorities. This aimed to increase flexibility, responsiveness, and local control over the education system, hoping to improve administrative efficiency (e.g., states can raise money for their schools in addition to federal support). These goals, along with the standardization and modernization of a national curriculum, were supported through the passage of the *Ley General de Educación* and *Acuerdo Nacional para la Modernización de la Educación Básica*. These reforms also sought to promote social participation (e.g., through the formation of the Consejos de Participación Social), involving the students, parents, teacher personnel, municipalities, and states in educational management and its quality improvement.^6–8^ Public education expenditures increased during this period. However, during the early 1990s, there were other financial reforms and privatization efforts intended to promote economic growth and development, including Mexico’s participation in the North American Free Trade Agreement.

Some scholars argue that the accelerated economic agenda under Salinas hindered a substantial impact on improving education quality and expanding equitable access across the country. Evidence suggests that gains in educational attainment have been gradual since the amendment of compulsory schooling. In 1993, the average educational attainment of the population aged 15 and older was 6.8 years; 10 years later, this increased to 7.9 years.^9^ Other scholars found that individuals exposed to changes from the 1993 Constitutional Amendment were more than twice as likely to transition from primary school to lower secondary school, demonstrating that the reform was effective at raising population-level educational attainment.^2^ In addition, one recent study found that exposure to the 1993 reform increased the average years of schooling by 0.7 years for an average child in the sample.^10^ Despite improvements at the national level, it is important to consider gender, urban-rural, socioeconomic, and regional variations in education expansion to comprehensively assess the impact of the 1993 Constitutional Amendment.

MHAS 2012 total sample of direct, proxy, & next of kin respondents

(n=15,723)

1,275 proxy respondents

794 direct respondents < 50 years

2012 direct respondents, aged ≥ 50

(n =13,654)

577 with no living children or missing information on total number of kids

5,372 oldest children were born more than 10 years before or more than 10 years after the CSL instrument

215 missing information on psychosocial outcomes

36 missing information on schooling of adult children

268 missing information on any of the covariates

Final analytical sample

(n=7,186)

Direct respondents with their oldest adult child impacted by the CSL

(n=7,705)

**eFigure 1.** Flowchart depicting analytic sample selection

**eAppendix 1. Description of psychosocial well-being measures and covariates**

*Psychosocial well-being measures*

Past-week depressive symptoms were assessed at each wave using a nine-item modified Center for Epidemiologic Studies Depression Scale.^11^ Respondents reported if they experienced the following symptoms for a majority of the time in the past week: 1) feeling depressed, 2) feeling that everything they did was difficult, 3) feeling they had restless sleep, 4) feeling happy (reverse coded), 5) feeling alone, 6) feeling that they enjoyed life (reverse coded), 7) feeling sad, 8) feeling tired, 9) feeling very energetic (reverse coded). Items were reported as binary “yes/no” response and summary counts ranged from 0 to 9 with higher values indicating more depressive symptoms.

Life satisfaction was assessed using an adapted version of the Satisfaction with Life Scale (SWLS).^12^ The adapted questionnaire used the same 5 items as the original scale, which are related to whether the respondent believed their life is close to their ideal, their life conditions are excellent, they are satisfied with life, they have gotten things that are important to them in life, and they would change almost nothing in their life, but modified the response options to three (vs. five) response options: 1 = Agree, 2 = Neither agree nor disagree, and 3 = Disagree. As each item is scored from 1 to 3, the possible range of scores of the adapted SWLS scale included in the MHAS is from 5 (high satisfaction) to 15 (low satisfaction).

*Covariates*

We controlled for respondents’ demographic characteristics including age (linear, cubic terms), gender (women, men), and marital status (married, widowed, single/divorced/separated). We additionally included measures of respondents’ own lifecourse SES. This included respondents’ own educational attainment, whether they spoke an indigenous dialect and the highest education level among their own parents, which may be an important marker of early-life SES. To capture other dimensions of respondents’ early life socioeconomic conditions we created a childhood disadvantage score. Respondents were asked if before the age of 10, they experienced any of the following: did not have access to sanitation facilities, generally went to bed hungry, did not regularly wear shoes, self/siblings had to drop out of school to support family, self/family slept in the kitchen, family received financial support, had a serious health problem that affected their normal activities. The disadvantage score (0-7) was generated from a summary count of their responses to the seven items, with higher values indicating higher disadvantage. Other lifecourse SES measures included respondents’ primary lifetime occupation (never worked for pay, white collar, blue collar, agricultural) created using groupings based on the Mexican Classification of Occupations;^13^ we included a missing indicator for those without lifetime occupation information, as occupation nonresponse could be indicative of lifetime socioeconomic disadvantage. If a respondent was married or partnered, we also included the following characteristics of the respondents’ spouses, the spouse’s baseline age, level of educational attainment, and lifetime occupation. Missing indicators were included for those who were not married or partnered. We did not adjust for measures of respondents’ current SES, such as wealth, income, and urbanicity, given that they could be potential mediators (downstream effects) of adult child’s education and could induce bias in our estimates.

**eAppendix 2. Description of the assumptions for instrumental variables analysis**

We invoked conventional assumptions for instrumental variables (IV) analysis, including relevance (i.e., the instrument (change in CSL) has a causal effect on the exposure (adult child education)), exclusion restriction (i.e., the CSL is only associated with parental psychosocial well-being through increases in adult child schooling), independence (i.e., the CSL and parental psychosocial well-being have no shared common causes after accounting for measured confounders), and monotonicity (i.e., no adult children achieved below the minimum levels of schooling because of the CSL or that were no ‘defiers’ of the CSL).^14,15^

We formally assessed the validity of the instrument in terms of the relevance assumption, by examining the relationship between the CSL and adult child schooling via the Kleibergen-Paap Wald F-statistic test^16^ and evaluate the F-statistic using the conventional cutoff of greater than 10 as the conventional cutoff.^17^ Nevertheless, this only establishes an association between the CSL and the exposure, rather than a causal relationship.

The rest of the assumptions cannot be formally tested and need to be theoretically defensible. Because parental sociodemographic characteristics may be correlated with the psychosocial wellbeing of older parents, our models also control for these variables, so that variation in the instrument comes from presumably exogenous (independent) differences in the CSL reform, and not from compositional differences in the sociodemographic characteristics of parents. This approach assumes that conditional on these characteristics, variation in the timing of the CSL reform is exogenous to the psychosocial wellbeing of older parents. In addition, although we cannot rule out that the instrument may pick up some of the age and period effects (i.e., other sociocontextual policies) on psychosocial wellbeing, our models control for parental age and age-squared and we have place birth cohort bounds to minimize potential violations of the exclusion restriction and independence assumptions.

The monotonicity assumption is plausible in our setting because in­creases in mandatory schooling would have likely not led respondents’ children to have less schooling than they would otherwise have completed in the absence of the CSL. Under these assumptions, the IV estimate is interpretable as the effect of additional schooling completed by the oldest child among those whose children increased schooling as a result of the change in CSL (i.e. the compliers).

| A) |
| --- |
| B) |

**eFigure 2.** Graphs for the mean psychosocial well-being scores of older Mexican adults (50+), by the child’s birth cohort. Plot of the mean A) depressive symptoms and B) life satisfaction score of older parents surrounding the birth cohorts of adult children impacted by the change in compulsory schooling laws. Depressive symptoms were coded such that higher scores reflected greater depressive symptomatology, with scores ranging from 0-9. Life satisfaction was coded such that higher scores reflected lower quality of life, with scores ranging from 5-15.

| **eTable 1.**  **Comparison of Beta Coefficients and 95% Confidence Intervals from Ordinary Least Squares Evaluating the Association between Exposure to the Compulsory Schooling Law Reform and Older Parents’ Psychosocial Outcomes in Mexico (n=7186)** | | |
| --- | --- | --- |
|  | OLS | |
|  | ß | 95% CI |
| Depressive symptoms (0-9) | -0.16 | (-0.30, -0.01) |
| Life satisfaction score (5-15)^a^ | 0.01 | (-0.14, 0.15) |
| Source: Mexican Health and Aging Study, 2012. Note: Reduced form estimates. Controls: Age, gender, marital status, own education, whether the respondent speaks an indigenous dialect, highest parental education level with missing indicator, early-life disadvantage score, own occupation, spousal age, spousal occupation, spousal education; models cluster standard errors at the household level. *a* Items were coded such that higher scores reflected lower quality of life. | | |

| 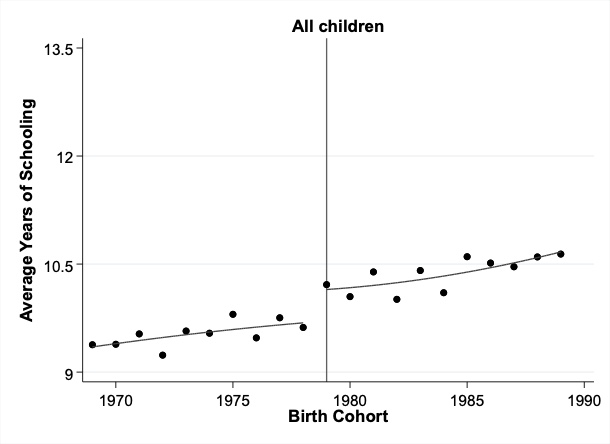A) |
| --- |
| 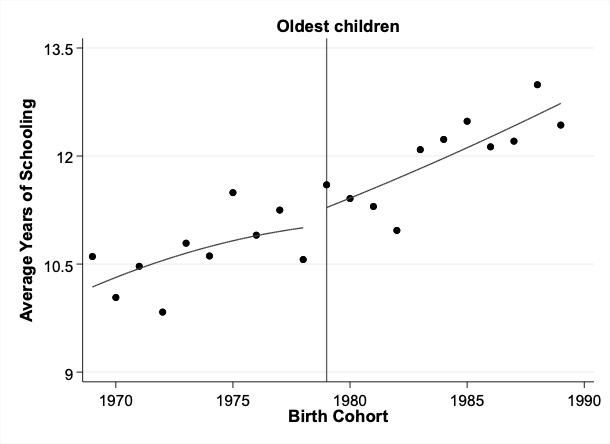B) |
| 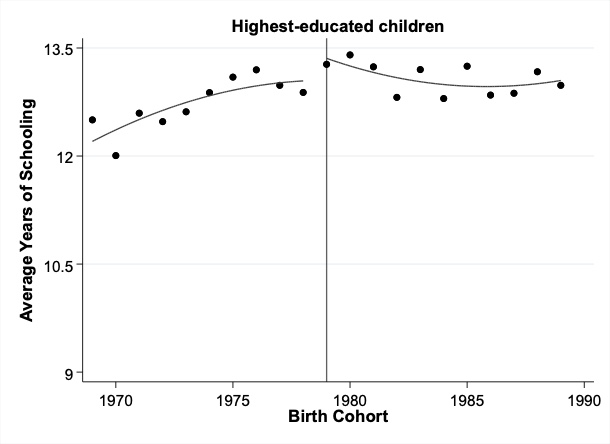C) |

**eFigure 3.** Graphs for the average educational attainment of the children of older Mexican adults (50+), by the child’s birth cohort. Average years of schooling by cohort are calculated for A) all children and B) the highest educated children, respectively.

| **eTable 2.**  **First-Stage Linear Probability Models of the Schooling Completion Thresholds of the Oldest Child on the Birth Cohort-Based Instrument and Kleibergen-Paap Wald F-Tests (n= 7186)** | | |
| --- | --- | --- |
|  | Coef. (95% CI) | *F* -statistic |
| At least 6 years of schooling | 0.013 (-0.003 – 0.028) | 2.47 |
| At least 7 years of schooling | 0.027 (0.003 – 0.052) | 4.90 |
| At least 8 years of schooling | 0.027 (0.002 – 0.052) | 4.56 |
| At least 9 years of schooling | 0.037 (0.010 – 0.064) | 7.40 |
| At least 10 years of schooling | 0.095 (0.064 – 0.125) | 36.68 |
| At least 11 years of schooling | 0.088 (0.058 – 0.119) | 31.72 |
| At least 12 years of schooling | 0.076 (0.046 – 0.107) | 23.73 |
| Source: Mexican Health and Aging Study, 2012. Note: Coefficients interpreted as the increased probability of having at least the threshold years of schooling. Controls: Age, gender, marital status, own education, whether the respondent speaks an indigenous dialect, highest parental education level with missing indicator, early-life disadvantage score, own occupation, spousal age, spousal occupation, spousal education | | |

| **eTable 3. Comparison of Beta Coefficients and 95% Confidence Intervals from Ordinary Least Squares and Two-Stage Least Squares Regressions Evaluating the Association between Oldest Daughter’s Educational Attainment and Older Parents’ Psychosocial Outcomes in Mexico, according to parent gender (n = 6725)** | | | | | | | | |
| --- | --- | --- | --- | --- | --- | --- | --- | --- |
|  | **Fathers (n= 2970)** | | | | **Mothers (n= 3755)** | | | |
|  | OLS | | 2SLS | | OLS | | 2SLS | |
|  | ß | [95% CI] | ß | [95% CI] | ß | [95% CI] | ß | [95% CI] |
| Depressive symptoms  (0-9) | -0.01 | (-0.04, 0.01) | 0.09 | (-0.18, 0.36) | -0.03 | (-0.05, -0.01) | -0.13 | (-0.34, 0.08) |
| Life satisfaction score  (5-15) ^a^ | -0.01 | (-0.03, 0.01) | 0.15 | (-0.11, 0.41) | -0.03 | (-0.06, -0.01) | 0.05 | (-0.15, 0.24) |
| Source: Mexican Health and Aging Study, 2012. Controls: Age, gender, marital status, own education, whether the respondent speaks an indigenous dialect, highest parental education level with missing indicator, early-life disadvantage score, own occupation, spousal age, spousal occupation, spousal education; models cluster standard errors at the household level. *a* Items were coded such that higher scores reflected lower quality of life. | | | | | | | | |

| **eTable 4. Comparison of Beta Coefficients and 95% Confidence Intervals from Ordinary Least Squares and Two-Stage Least Squares Regressions Evaluating the Association between Oldest Son's Educational Attainment and Older Parents’ Psychosocial Outcomes in Mexico, according to parent gender (n = 6707)** | | | | | | | | |
| --- | --- | --- | --- | --- | --- | --- | --- | --- |
|  | **Fathers (n= 2998)** | | | | **Mothers (n= 3709)** | | | |
|  | OLS | | 2SLS | | OLS | | 2SLS | |
|  | ß | [95% CI] | ß | [95% CI] | ß | [95% CI] | ß | [95% CI] |
| Depressive symptoms (0-9) | -0.05 | (-0.07, -0.02) | -0.29 | (-0.88, 0.30) | -0.04 | (-0.06, -0.02) | -0.42 | (-0.82, -0.02) |
| Life satisfaction score (5-15) ^a^ | -0.05 | (-0.07, -0.03) | 0.49 | (-0.21, 1.19) | -0.04 | (-0.06, -0.02) | 0.10 | (-0.23, 0.43) |
| Source: Mexican Health and Aging Study, 2012. Controls: Age, gender, marital status, own education, whether the respondent speaks an indigenous dialect, highest parental education level with missing indicator, early-life disadvantage score, own occupation, spousal age, spousal occupation, spousal education; models cluster standard errors at the household level. *a* Items were coded such that higher scores reflected lower quality of life. | | | | | | | | |

| **eTable 5. Comparison of Beta Coefficients and 95% Confidence Intervals from Two-Stage Least Squares Regressions Evaluating the Association between Oldest Adult Children's Educational Attainment and Psychosocial Outcomes for Older Parents aged 50+ in Mexico, by Coresidence Status** | | | | | | |
| --- | --- | --- | --- | --- | --- | --- |
| **Panel A: First-stage results** | | | | | | |
|  | Oldest Child Co-resides  (n=2152) | | | Oldest Child Does Not Co-reside  (n= 5034) | | |
|  | ß | [95% CI] | F | ß | [95% CI] | F |
| Average schooling of oldest child | 0.45 | (-0.00, 0.90) | 3.84 | 0.53 | (0.22, 0.83) | 11.48 |
| **Panel B: Second-stage results** | | | | | | |
|  | Oldest Child Co-resides  (n=2152) | | | Oldest Child Does Not Co-reside  (n= 5034) | | |
|  | ß | [95% CI] |  | ß | [95% CI] |  |
| Depressive symptoms (0-9) | -- |  |  | -0.26 | (-0.64, 0.11) |  |
| Life satisfaction score (5-15) ^a^ | -- |  |  | -0.01 | (-0.35, 0.33) |  |
| Source: Mexican Health and Aging Study, 2012. Only second-stage results meeting the first-stage F-statistic above the conventional cut-off of 10 are shown. Controls: Age, gender, marital status, own education, whether the respondent speaks an indigenous dialect, highest parental education level with missing indicator, early-life disadvantage score, own occupation, spousal age, spousal occupation, spousal education; models cluster standard errors at the household level. *a* Items were coded such that higher scores reflected lower quality of life. | | | | | | |

| **eTable 6. Comparison of Beta Coefficients and 95% Confidence Intervals from Two-Stage Least Squares Regressions Evaluating the Association between Oldest Adult Children's Educational Attainment and Psychosocial Outcomes for Older Parents aged 50+ in Mexico, by Parents' Educational Attainment** | | | | | | |
| --- | --- | --- | --- | --- | --- | --- |
| **Panel A: First-stage results** | | | | | | |
|  | Parent has 0-5 years of education  (n=2666) | | | Parent at least completed primary school, 6+ years (n=4520) | | |
|  | ß | [95% CI] | F | ß | [95% CI] | F |
| Average schooling of oldest child | 0.28 | (-0.12, 0.68) | 1.86 | 0.84 | (0.55, 1.13) | 32.10 |
| **Panel B: Second-stage results** | | | | | | |
|  | Parent has 0-5 years of education  (n=2666) | | | Parent at least completed primary school, 6+ years (n=4520) | | |
|  | ß | [95% CI] |  | ß | [95% CI] |  |
| Depressive symptoms (0-9) | -- |  |  | -0.16 | (-0.37, 0.06) |  |
| Life satisfaction score (5-15) ^a^ | -- |  |  | -0.02 | (-0.25, 0.20) |  |
| Source: Mexican Health and Aging Study, 2012. Only second-stage results meeting the first-stage F-statistic above the conventional cut-off of 10 are shown. Controls: Age, gender, marital status, own education, whether the respondent speaks an indigenous dialect, highest parental education level with missing indicator, early-life disadvantage score, own occupation, spousal age, spousal occupation, spousal education; models cluster standard errors at the household level. *a* Items were coded such that higher scores reflected lower quality of life. | | | | | | |

| **eTable 7. Comparison of Beta Coefficients and 95% Confidence Intervals from Two-Stage Least Squares Regressions Evaluating the Association between Oldest Adult Children's Educational Attainment and Psychosocial Outcomes for Older Parents aged 50+ in Mexico, by Urbanicity** | | | | | | |
| --- | --- | --- | --- | --- | --- | --- |
| **Panel A: First-stage results** | | | | | | |
|  | Respondent doesn't live in urban locality (n=2812) | | | Respondent lives in urban locality (n=4374) | | |
|  | ß | [95% CI] | F | ß | [95% CI] | F |
| Average schooling of oldest child | 0.49 | (0.08, 0.89) | 5.55 | 0.79 | (0.48, 1.09) | 25.30 |
| **Panel B: Second-stage results** | | | | | | |
|  | Respondent doesn't live in urban locality (n=2812) | | | Respondent lives in urban locality (n=4374) | | |
|  | ß | [95% CI] |  | ß | [95% CI] |  |
| Depressive symptoms (0-9) | -- |  |  | -0.27 | (-0.53, -0.01) |  |
| Life satisfaction score (5-15) ^a^ | -- |  |  | -0.04 | (-0.29, 0.21) |  |
| Source: Mexican Health and Aging Study, 2012. Only second-stage results meeting the first-stage F-statistic above the conventional cut-off of 10 are shown. Controls: Age, gender, marital status, own education, whether the respondent speaks an indigenous dialect, highest parental education level with missing indicator, early-life disadvantage score, own occupation, spousal age, spousal occupation, spousal education; models cluster standard errors at the household level. *a* Items were coded such that higher scores reflected lower quality of life. | | | | | | |

| **eTable 8. The association of exposure to CSL reform on the schooling of the highest educated adult child (IV 2SLS first-stage) and the association of the highest educated child’s schooling on psychosocial outcomes (IV 2SLS second-stage and OLS) (n=8,196)** | | | | | |
| --- | --- | --- | --- | --- | --- |
|  | **Method 1: OLS** | | **Method 2: 2SLS** | |  |
| Depressive symptoms | ß | (95% CI) | ß | (95% CI) |  |
| Association of each year increase in educational attainment of highest educated adult child on depressive symptoms | -0.03 | (-0.05, -0.01) |  |  |  |
| First-stage: association of exposure to CSL on educational attainment of highest educated adult child |  |  | 0.41 | (0.21, 0.61) |  |
| Second-stage: association of the predicted value of educational attainment of highest educated adult child on depressive symptoms |  |  | -0.16 | (-0.48, 0.16) |  |
| Life satisfaction score |  |  |  |  |  |
| Association of each year increase in educational attainment of highest educated adult child on life satisfaction score | -0.04 | (-0.06, -0.03) |  |  |  |
| First-stage: association of exposure to CSL on educational attainment of highest educated adult child |  |  | 0.41 | (0.21, 0.61) |  |
| Second-stage: association of the predicted value of educational attainment of highest educated adult child on life satisfaction |  |  | -0.06 | (-0.35, 0.23) |  |
| Source: Mexican Health and Aging Study, 2012. Controls: Age, gender, marital status, own education, whether the respondent speaks an indigenous dialect, highest parental education level with missing indicator, early-life disadvantage score, own occupation, spousal age, spousal occupation, spousal education; models cluster standard errors at the household level. Life satisfaction items were coded such that higher scores reflected lower quality of life. | | | | | |

| **eTable 9. Comparison of Beta Coefficients and 95% Confidence Intervals from Ordinary Least Squares and Two-Stage Least Squares Regressions Evaluating the Association between the Highest Educated Adult Child’s Schooling and Older Parents’ Psychosocial Outcomes in Mexico, by parent gender** | | | | | | | | | |
| --- | --- | --- | --- | --- | --- | --- | --- | --- | --- |
|  | **Fathers (n= 3599)** | | | | **Mothers (n= 4597)** | | | | |
|  | OLS | | 2SLS | | OLS | | 2SLS | | |
|  | ß | [95% CI] | ß | [95% CI] | ß | [95% CI] | ß | [95% CI] |  |
| Depressive symptoms (0-9) | -0.03 | (-0.05, -0.00) | -0.08 | (-0.48, 0.32) | -0.03 | (-0.06, -0.01) | -0.20 | (-0.63, 0.22) |  |
| Life satisfaction score (5-15) | -0.03 | (-0.06, -0.01) | -0.08 | (-0.45, 0.30) | -0.05 | (-0.07, -0.02) | -0.01 | (-0.39, 0.37) |  |
| Source: Mexican Health and Aging Study, 2012. Controls: Age, gender, marital status, own education, whether the respondent speaks an indigenous dialect, highest parental education level with missing indicator, early-life disadvantage score, own occupation, spousal age, spousal occupation, spousal education; models cluster standard errors at the household level. Life satisfaction items were coded such that higher scores reflected lower quality of life. | | | | | | | | | |

| **eTable 10. The association of exposure to CSL reform (instrument: average required schooling) on the average educational attainment of all children 25 years or older (IV 2SLS first-stage) and the association of average educational attainment of all children (25+) on psychosocial outcomes (IV 2SLS second-stage and OLS)** | | | | |
| --- | --- | --- | --- | --- |
| **Panel A: First-stage 2SLS estimates** | |  |  |  |
|  | n | ß | [95% CI] | F |
| Years of schooling, all children (25+) | 11059 | 0.13 | 0.05, 0.22 | 9.44 |
|  |  |  |  |  |
| **Panel B: OLS and second-stage 2SLS estimates** | | | | |
|  | **Average Schooling of All Children, 25+ (n=11059)** | | | |
|  | OLS | | 2SLS | |
|  | ß | [95% CI] | ß | [95% CI] |
| Depressive symptoms (0-9) | -0.06 | (-0.08, -0.05) | -0.21 | (-0.69, 0.26) |
| Life satisfaction score (5-15) ^a^ | -0.06 | (-0.07, -0.04) | 0.48 | (-0.07, 1.04) |
| Source: Mexican Health and Aging Study, 2012. Controls: Age, gender, marital status, own education, whether the respondent speaks an indigenous dialect, highest parental education level with missing indicator, early-life disadvantage score, own occupation, spousal age, spousal occupation, spousal education; models cluster standard errors at the household level. *a* Items were coded such that higher scores reflected lower quality of life. | | | | |

| **eTable 11. First-Stage 2SLS estimates and Kleibergen-Paap Wald F-Tests for Alternate Bound Specifications Birth Cohort-Based Instrumental Variable on Years of Oldest Adult Child Schooling** | | | | |
| --- | --- | --- | --- | --- |
|  | n | ß | [95% CI] | F |
| 5-year bounds | 4230 | 0.38 | 0.09, 0.66 | 6.79 |
| 15-year bounds | 9030 | 0.60 | 0.36, 0.83 | 24.35 |
| Source: Mexican Health and Aging Study, 2012. Controls: Age, gender, marital status, own education, whether the respondent speaks an indigenous dialect, highest parental education level with missing indicator, early-life disadvantage score, spousal age, spousal occupation, spousal education; models cluster standard errors at the household level. | | | | |

| **eTable 12. Comparison of Beta Coefficients and 95% Confidence Intervals from Two-Stage Least Squares Regressions Evaluating the Association between Oldest Child's Educational Attainment and Older Parents’ Psychosocial Outcomes in Mexico, by instrument bound type** | | | | |
| --- | --- | --- | --- | --- |
|  | **Oldest adult child** | | | |
|  | 5-year bounds | | 15-year bounds | |
|  | ß | [95% CI] | ß | [95% CI] |
| Depressive symptoms (0-9) | -0.26 | (-0.73, 0.21) | -0.24 | (-0.47, -0.01) |
| Life satisfaction score (5-15) | -0.21 | (-0.66, 0.24) | 0.03 | (-0.19, 0.24) |
| Source: Mexican Health and Aging Study, 2012. Controls: Age, gender, marital status, own education, whether the respondent speaks an indigenous dialect, highest parental education level with missing indicator, early-life disadvantage score, spousal age, spousal occupation, spousal education; models cluster standard errors at the household level. Life satisfaction items were coded such that higher scores reflected lower quality of life. | | | | |

| **eTable 13. Beta Coefficients and 95% Confidence Intervals from Two-Stage Least Squares Regressions Evaluating the Association between the Educational Attainment of the Oldest Child and Older Parents’ Psychosocial Outcomes in Mexico, using a 2-year wash out period** | | | | |
| --- | --- | --- | --- | --- |
| **Panel A: First-stage 2SLS estimates** | |  |  |  |
|  | n | ß | [95% CI] | F |
| Years of schooling, oldest child | 6542 | 0.70 | 0.42, 0.98 | 24.02 |
|  |  |  |  |  |
| **Panel B: Second-stage 2SLS estimates** | | | | |
|  | **Educational Attainment of the Oldest Child (n=11062)** | | | |
|  | 2SLS | |  | |
|  | ß | [95% CI] |  |  |
| Depressive symptoms (0-9) | -0.22 | (-0.46, 0.02) |  |  |
| Life satisfaction score (5-15) ^a^ | 0.02 | (-0.21, 0.25) |  |  |
| Source: Mexican Health and Aging Study, 2012. Controls: Age, gender, marital status, own education, whether the respondent speaks an indigenous dialect, highest parental education level with missing indicator, early-life disadvantage score, own occupation, spousal age, spousal occupation, spousal education; models cluster standard errors at the household level. *a* Items were coded such that higher scores reflected lower quality of life. | | | | |

| **eTable 14.**  **Comparison of Coefficients and 95% Confidence Intervals from Logit and Two-Stage Least Squares Regression Evaluating the Association between Oldest Child’s Educational Attainment and Older Parents’ Elevated Depressive Symptomatology in Mexico, Overall and by Gender** | | | | | | | | | | |
| --- | --- | --- | --- | --- | --- | --- | --- | --- | --- | --- |
| **Panel A: Overall** | | | | | | | | | | |
|  | **Oldest Child (n=7186)** | | | | |  |  |  |  |  |
|  | Logit | | 2SLS | | |  |  |  |  |  |
|  | Coef. | 95% CI | Coef. | 95% CI | |  |  |  |  |  |
| Elevated depressive symptoms ^a^ | -0.04 | (-0.06, -0.03) | -0.12 | (-0.23, -0.00) | |  |  |  |  |  |
| **Panel B: Parent’s Gender** | | | | | | | | | | |
|  | **Fathers (n= 3238)** | | | | |  | **Mothers (n= 3948)** | | | |
|  | Logit | | 2SLS | | |  | Logit | | 2SLS | |
|  | Coef. | 95% CI | Coef. | | 95% CI |  | Coef. | 95% CI | Coef. | 95% CI |
| Elevated depressive symptoms ^a^ | -0.05 | (-0.07, -0.02) | -0.21 | | (-0.35, 0.07) |  | -0.04 | (-0.06, -0.02) | -0.07 | (-0.20, 0.06) |
| **Panel C: Adult Child’s Gender** | | | | | | | | | | |
|  | **Oldest Daughter (n=6725)** | | | | |  | **Oldest Son (n=6707)** | | | |
|  | Logit | | 2SLS | | |  | Logit | | 2SLS | |
|  | Coef. | 95% CI | Coef. | | 95% CI |  | Coef. | 95% CI | Coef. | 95% CI |
| Elevated depressive symptoms ^a^ | -0.03 | (-0.04, -0.01) | -0.02 | | (-0.12, 0.07) |  | -0.05 | (-0.06, -0.03) | -0.14 | (-0.28, -0.00) |
| Source: Mexican Health and Aging Study, 2012. Estimates should be interpreted as log-odds for logit models. Controls: Age, gender, marital status, own education, whether the respondent speaks an indigenous dialect, highest parental education level with missing indicator, early-life disadvantage score, own occupation, spousal age, spousal occupation, spousal education; models cluster standard errors at the household level. *a* Participants with CES-D scores of 5 or more were categorized as having elevated depressive symptomatology. The reference group was low or no depressive symptomatology (0 to 4 CES-D score). | | | | | | | | | | |

**References for Supplemental Material**

1. Pérez Sánchez S. Educación laica en el sistema educativo mexicano: entre la omisión, la ambigüedad y el conflicto. *Páginas Educ*. 2012;5(1):79-95.

2. Creighton M, Park H. Closing the Gender Gap: Six Decades of Reform in Mexican Education. *Comp Educ Rev*. 2010;54(4):513-537. doi:10.1086/653702

3. Gill CC. *Education in a Changing Mexico*.; 1969. Accessed January 23, 2024. https://eric.ed.gov/?id=ED054024

4. Bentaouet Kattan R, Székely M. Patterns, Consequences, and Possible Causes of Dropout in Upper Secondary Education in Mexico. *Educ Res Int*. 2015;2015:e676472. doi:10.1155/2015/676472

5. Navarro-Leal MA, Navarrete-Cazales Z. The Mexican Education Reforms and the Teacher Education System at the Turn of the Century. Published online 2014. http://archive.org/details/ERIC_ED598068

6. Ley General de Educación. https://www.sep.gob.mx/work/models/sep1/Resource/3f9a47cc-efd9-4724-83e4-0bb4884af388/ley_general_educacion.pdf

7. RAMO GENERAL 33. FONDO DE APORTACIONES PARA LA EDUCACIÓN BÁSICA Y NORMAL (FAEB). https://www.asf.gob.mx/Trans/Informes/IR2009i/Tomos/Tomo5/01_FAEB_a.pdf

8. Reforma 2019 a los artículos 3°, 31 y 73 de la Constitución Política de los Estados Unidos Mexicanos. *Perfiles Educ*. 2019;41(165):186-208. doi:10.22201/iisue.24486167e.2019.165.59496

9. Santibañez L, Vernez G, Razquin P. *Education in Mexico: Challenges and Opportunities*. RAND Corporation; 2005.

10. Ma M, Yahirun J, Saenz J, Sheehan C. Offspring Educational Attainment and Older Parents’ Cognition in Mexico. *Demography*. 2021;(8931725). doi:10.1215/00703370-8931725

11. Radloff LS. The CES-D Scale: A Self-Report Depression Scale for Research in the General Population. *Appl Psychol Meas*. 1977;1(3):385-401. doi:10.1177/014662167700100306

12. López-Ortega M, Torres-Castro S, Rosas-Carrasco O. Psychometric properties of the Satisfaction with Life Scale (SWLS): secondary analysis of the Mexican Health and Aging Study. *Health Qual Life Outcomes*. 2016;14:170. doi:10.1186/s12955-016-0573-9

13. INEGI. *Clasificación mexicana de ocupaciones. Volumen I*. INEGI; 1994.

14. Hernan MA, Robins JM. Instrumental variable estimation. In: *Causal Inference: What If*. Chapman & Hall/CRC Press; 2023:203-220. https://www.hsph.harvard.edu/miguel-hernan/wp-content/uploads/sites/1268/2023/10/hernanrobins_WhatIf_30sep23.pdf

15. Labrecque J, Swanson SA. Understanding the Assumptions Underlying Instrumental Variable Analyses: a Brief Review of Falsification Strategies and Related Tools. *Curr Epidemiol Rep*. 2018;5(3):214-220. doi:10.1007/s40471-018-0152-1

16. Kleibergen F, Paap R. Generalized reduced rank tests using the singular value decomposition. *J Econom*. 2006;133(1):97-126. doi:10.1016/j.jeconom.2005.02.011

17. Stock JH, Yogo M. Testing for Weak Instruments in Linear IV Regression. Published online November 2002. doi:10.3386/t0284
